# Early ΔNLR Outperforms Baseline Inflammatory Markers in Predicting Short-Term Outcomes in Sepsis

**DOI:** 10.3390/diagnostics16101473

**Published:** 2026-05-12

**Authors:** Madalina-Ianca Suba, Gheorghe-Bogdan Hogea, Varga Norberth-Istvan, Florina Cristiana Lucaciu, Camelia Corina Pescaru, Ovidiu Rosca, Daniela Gurgus, Bogdan Rotea, Andra Rotea, Ahmed Abu-Awwad, Anca Mihaela Bina, Daniel Pop, Simona-Alina Abu-Awwad

**Affiliations:** 1 Methodological and Infectious Diseases Research Center, Faculty of Medicine, “Victor Babeș” University of Medicine and Pharmacy Timisoara, 300041 Timisoara, Romania; madalina.suba@umft.ro (M.-I.S.); ovidiu.rosca@umft.ro (O.R.); 2 Pulmonary Rehabilitation Center, Clinical Hospital of Infectious Diseases and Pulmonology “Victor Babes”, Gheorghe Adam Street 13, 300310 Timisoara, Romania; florina.lucaciu@umft.ro (F.C.L.); pescaru.camelia@umft.ro (C.C.P.); 3 ”Pius Brinzeu” Emergency Clinical County Hospital, Bld Liviu Rebreanu, No. 156, 300723 Timisoara, Romania; hogea.bogdan@umft.ro (G.-B.H.); lungu.anca@umft.ro (A.M.B.); alina.abuawwad@umft.ro (S.-A.A.-A.); 4 Department XV, Discipline of Orthopedics-Traumatology, “Victor Babeș” University of Medicine and Pharmacy Timisoara, Eftimie Murgu Square, No. 2, 300041 Timisoara, Romania; daniel.pop@umft.ro; 5 Profesor Universitar Doctor Teodor Sora Research Centre, “Victor Babeș” University of Medicine and Pharmacy Timisoara, Eftimie Murgu Square, No. 2, 300041 Timisoara, Romania; 6 Department of Nursing, “Victor Babeș” University of Medicine and Pharmacy Timisoara, Eftimie Murgu Square, No. 2, 300041 Timisoara, Romania; norberth.varga@umft.ro; 7 Doctoral School, “Victor Babeș” University of Medicine and Pharmacy Timisoara, 2 Eftimie Murgu Square, 300041 Timisoara, Romania; bogdan.rotea@umft.ro (B.R.); andra.rotea@umft.ro (A.R.); 8 Center for Research and Innovation in Personalized Medicine of Respiratory Diseases (CRIPMRD), “Victor Babeș” University of Medicine and Pharmacy Timisoara, Eftimie Murgu Square 2, 300041 Timisoara, Romania; 9 Department of Balneology, Medical Recovery and Rheumatology, Family Discipline, Center for Preventive Medicine, “Victor Babeș” University of Medicine and Pharmacy Timisoara, 300041 Timisoara, Romania; 10 Department of Obstetrics and Gynecology, “Victor Babeș” University of Medicine and Pharmacy Timisoara, 300041 Timisoara, Romania

**Keywords:** sepsis, neutrophil-to-lymphocyte ratio, NLR, inflammatory biomarkers, dynamic biomarkers, risk stratification, SOFA score, prognosis, in-hospital mortality, critical care

## Abstract

**Background/Objectives**: Sepsis is a dynamic clinical syndrome characterized by a rapidly evolving inflammatory response, where early identification of patients at risk for adverse outcomes remains a major challenge. While inflammatory biomarkers are widely used, their prognostic value at baseline is limited. This study aimed to evaluate whether early changes in inflammatory biomarkers, particularly the neutrophil-to-lymphocyte ratio (ΔNLR), provide additional prognostic value in predicting short-term outcomes in patients with sepsis. **Methods**: A retrospective longitudinal observational study was conducted, including 168 adult patients admitted with sepsis at a tertiary infectious diseases hospital. Inflammatory biomarkers (CRP, procalcitonin, leukocyte subpopulations, and NLR) were assessed at admission and at 48–72 h. Early changes (Δ values) were calculated and analyzed in relation to a composite adverse outcome, including ICU admission, vasopressor requirement, mechanical ventilation, or in-hospital mortality. Logistic regression and ROC curve analyses were used to evaluate predictive performance. **Results**: Patients with adverse outcomes had significantly higher baseline inflammatory markers and severity scores. Early reductions in CRP and NLR were more pronounced in survivors, whereas non-survivors showed persistently elevated or minimally decreasing values. In multivariate analysis, ΔNLR remained independently associated with in-hospital mortality (OR 0.91, 95% CI 0.84–0.98, *p* = 0.015), alongside Sequential Organ Failure Assessment (SOFA) score and septic shock. ΔNLR demonstrated better discriminative performance (AUC 0.74) compared to baseline markers and improved predictive accuracy when combined with SOFA score (AUC 0.81). Higher baseline NLR quartiles were associated with a stepwise increase in adverse outcomes. **Conclusions**: Early changes in inflammatory biomarkers, particularly ΔNLR, provide clinically relevant prognostic information beyond baseline measurements and severity scores in sepsis. Dynamic assessment of immune response may improve early risk stratification and support more individualized clinical decision-making.

## 1. Introduction

Sepsis continues to represent a major challenge in contemporary medicine, being one of the leading causes of in-hospital mortality and prolonged intensive care admissions worldwide [1]. Defined as a life-threatening organ dysfunction resulting from a dysregulated host response to infection, sepsis is not a static diagnosis but a rapidly evolving clinical syndrome [2]. Despite advances in antimicrobial therapy, organ support, and standardized management protocols, outcomes remain highly variable [3]. Some patients stabilize quickly under appropriate treatment, while others experience rapid deterioration, progressing to septic shock and multiple organ failure within a short timeframe [4].

At the core of sepsis lies a complex and dynamic inflammatory response. The initial interaction between the invading pathogen and the host immune system triggers the activation of innate immune pathways, leading to the release of pro-inflammatory cytokines, chemokines, and acute-phase proteins [5]. This cascade promotes leukocyte recruitment, endothelial activation, and coagulation pathway engagement [6]. While this response is essential for infection control, its amplification or persistence can result in endothelial dysfunction, capillary leak, tissue hypoperfusion, and cellular metabolic failure. Importantly, the inflammatory profile of a septic patient is not fixed at presentation; it changes over time, reflecting the balance between ongoing infection, therapeutic interventions, and individual immune regulation [7,8].

In routine clinical practice, inflammatory biomarkers play a central role in the diagnosis and monitoring of sepsis [9]. Parameters such as C-reactive protein (CRP) [10], procalcitonin (PCT) [11], total leukocyte count, neutrophil and lymphocyte subpopulations, and derived indices like the neutrophil-to-lymphocyte ratio (NLR) are widely available and frequently used [12]. The neutrophil-to-lymphocyte ratio (NLR) is a simple index calculated as the ratio between absolute neutrophil and lymphocyte counts in peripheral blood. From a pathophysiological perspective, it reflects the balance between innate immune activation, represented by neutrophilia, and adaptive immune suppression, reflected by lymphopenia, thus providing an integrated marker of immune response dysregulation [13].

These markers assist clinicians in evaluating the severity of infection, guiding antimicrobial decisions, and assessing response to treatment. However, clinical experience often suggests that the initial value alone does not fully capture the patient’s trajectory. Two patients may present with similar baseline biomarker levels, yet their subsequent evolution can differ substantially [14].

The early phase of sepsis, particularly the first 48 to 72 h, represents a critical therapeutic window. During this period, trends in inflammatory biomarkers may offer more meaningful information than isolated measurements. A rapid decline in inflammatory markers may reflect adequate infection control and favorable host response, whereas persistently elevated or rising values may signal ongoing inflammation, inadequate source control, or impending organ dysfunction. Evaluating these early changes could therefore enhance risk stratification and allow for more timely therapeutic adjustments [14,15,16].

Moreover, short-term outcomes in sepsis, including need for intensive care admission, requirement for vasopressor or ventilatory support, prolonged hospitalization, and in-hospital mortality, are closely linked to the early inflammatory burden and its resolution [17]. Identifying reliable, easily measurable predictors of these outcomes is of practical importance, especially in settings where rapid clinical decisions must be made based on limited information.

To our knowledge, this study is among the first real-world analyses to demonstrate that early changes in neutrophil-to-lymphocyte ratio (ΔNLR) provide independent and incremental prognostic value beyond baseline inflammatory markers and established severity scores, highlighting the clinical relevance of dynamic immune monitoring in sepsis.

In this context, analyzing the dynamic changes of inflammatory biomarkers during the early course of hospitalization may provide valuable insights into patient prognosis. Therefore, the aim of this study is to investigate whether early changes in inflammatory biomarkers are associated with short-term clinical outcomes in patients with sepsis and to identify which biomarker trajectories best predict adverse evolution during hospitalization.

Unlike prior studies focusing on static biomarkers or specific conditions such as COVID-19 [18,19], this study evaluates early inflammatory dynamics in a real-world sepsis population and demonstrates the independent prognostic value of ΔNLR beyond established severity scores.

## 2. Materials and Methods

### 2.1. Study Design and Setting

This study was designed as a retrospective longitudinal observational study conducted at the Clinical Hospital of Infectious Diseases and Pneumophthisiology “Dr. Victor Babeș”, Timișoara, Romania. The analysis was based on data extracted from the institutional electronic medical records and laboratory database of patients admitted with sepsis during the predefined study period.

According to the official ethical approval document, the study covered patients hospitalized between 1 July 2023 and 31 January 2026. The research protocol was reviewed and approved by the hospital management, the Data Protection Officer, and the Ethics Committee of the Clinical Hospital of Infectious Diseases and Pneumophthisiology “Dr. Victor Babeș” Timișoara (approval No. 935/2 February 2026).

The study was conducted in accordance with the Declaration of Helsinki and complied with Regulation (EU) No. 679/2016 (GDPR) regarding the protection of personal data, as explicitly stated in the ethical approval document.

Informed consent was obtained from all subjects involved in the study.. All patient data were anonymized before analysis. Access to identifiable information was restricted, and data were processed exclusively for research purposes, under strict confidentiality and data protection measures.

### 2.2. Study Population

The study included adult patients (≥18 years) admitted to the hospital with a diagnosis of sepsis during the study interval. Sepsis was defined as suspected or confirmed infection associated with acute organ dysfunction, according to contemporary clinical criteria documented in the medical records [20]. Pediatric patients were not included, as the study was conducted in an adult tertiary care center and aimed to evaluate a relatively homogeneous population with comparable immunological and clinical characteristics.

Patients were eligible if they met all the following criteria:

- Age ≥18 years.
- Hospitalization during the study period (1 July 2023–31 January 2026).
- Documented diagnosis of sepsis, defined as suspected or confirmed infection associated with acute organ dysfunction according to contemporary clinical criteria.
- Availability of inflammatory biomarker measurements at admission (within 24 h) and at least one repeat measurement within 48–72 h, enabling assessment of early biomarker kinetics.
- Complete documentation of predefined short-term in-hospital outcomes.
- Length of stay ≥24 h.

For patients with multiple eligible admissions, only the first hospitalization during the study period was included.

Patients were excluded if they had:

- Insufficient serial laboratory data to assess early inflammatory dynamics.
- Transfer from another institution after >48 h of treatment for the same infectious episode.
- Conditions known to substantially alter inflammatory or leukocyte profiles, including active hematologic malignancy, profound chronic neutropenia, recent cytotoxic chemotherapy, solid organ or hematopoietic stem cell transplantation, or ongoing high-dose immunosuppressive therapy.
- Major concurrent non-infectious inflammatory insults at admission (e.g., extensive trauma, major recent surgery, or large-surface burns).
- Missing or unreliable outcome data.

### 2.3. Data Collection

Data were retrospectively extracted from electronic medical records and hospital laboratory information systems. For each patient, the following variables were collected: Demographic characteristics (age, sex); Clinical parameters at admission, including suspected or confirmed source of infection and documented organ dysfunction; Laboratory inflammatory biomarkers measured at admission (baseline) and during the first 48–72 h of hospitalization.

The inflammatory biomarkers analyzed included C-reactive protein (CRP), procalcitonin (PCT), total leukocyte count, absolute neutrophil count, absolute lymphocyte count, and the neutrophil-to-lymphocyte ratio (NLR). Due to the non-normal distribution of NLR values, data were expressed as median and interquartile range, and comparisons were performed using non-parametric tests.

Early changes in inflammatory biomarkers were calculated as absolute differences and relative percentage changes between baseline values and follow-up measurements within the first 48–72 h. Based on these dynamics, patients were categorized according to biomarker evolution (decreasing vs. persistently elevated or increasing levels).

### 2.4. Outcomes

The primary outcome was a composite endpoint reflecting short-term in-hospital clinical deterioration, defined by the occurrence of at least one of the following: ICU admission, need for vasopressor support, requirement for invasive mechanical ventilation, or in-hospital death.

Secondary outcomes comprised progression to septic shock and hospital length of stay.

### 2.5. Statistical Analysis

Statistical analysis was performed using GraphPad Prism 10 (GraphPad Software, San Diego, CA, USA) and MedCalc version 23.3.2 (MedCalc Software Ltd., Ostend, Belgium). A two-sided *p*-value < 0.05 was considered statistically significant.

Continuous variables were expressed as mean ± standard deviation (SD) for approximately normally distributed data and as median with interquartile range (IQR) for non-normally distributed variables. Categorical variables were presented as absolute frequencies and percentages.

The distribution of continuous variables was assessed prior to analysis. Variables with non-normal distribution, including neutrophil-to-lymphocyte ratio (NLR) and procalcitonin (PCT), were analyzed using non-parametric methods. Comparisons between two groups were performed using the independent samples *t*-test for normally distributed continuous variables and the Mann–Whitney U test for non-normally distributed variables. Categorical variables were compared using the chi-square test or Fisher’s exact test, as appropriate.

Early changes in inflammatory biomarkers (Δ values) were calculated as the difference between baseline measurements (at admission) and follow-up values obtained within 48–72 h. These dynamic variables were analyzed both as continuous parameters and as part of regression models. To enhance robustness, both absolute and relative changes in biomarkers were evaluated in sensitivity analyses.

Univariate logistic regression analysis was performed to identify variables associated with in-hospital mortality. Variables with statistical significance or clinical relevance were subsequently included in multivariate logistic regression models to determine independent predictors. Results were reported as odds ratios (ORs) with 95% confidence intervals (CIs).

Receiver operating characteristic (ROC) curve analysis was conducted to evaluate the discriminative performance of baseline and dynamic inflammatory biomarkers. The area under the curve (AUC) with 95% confidence intervals was calculated, and optimal cut-off values were determined using the Youden index. Sensitivity and specificity were reported for each parameter. ROC analysis was restricted to variables with complete-case data across the study cohort to ensure robustness and comparability of predictive performance estimates.

Correlation analysis between inflammatory biomarkers and disease severity (Sequential Organ Failure Assessment (SOFA) score) [21] was performed using Spearman’s rank correlation coefficient, given the non-normal distribution of several variables.

All analyses were performed using complete-case data, and no imputation methods were applied for missing values.

## 3. Results

The study cohort included 168 patients with sepsis, predominantly elderly, with a moderate severity of organ dysfunction at admission. The distribution of infection sources and comorbidities reflected a typical septic population, with pulmonary and urinary infections being the most frequent. Detailed baseline characteristics are presented in Table 1.

A comparison between survivors and non-survivors revealed a clear association between disease severity and outcome. Patients who died during hospitalization had higher SOFA scores and a greater inflammatory burden at admission, particularly reflected by elevated CRP and NLR values (Table 2).

Similarly, Table 3 presents the characteristics of patients who developed at least one adverse in-hospital outcome showed significantly higher baseline severity scores and inflammatory markers, including CRP, procalcitonin, and NLR, while age did not differ significantly between groups (Table 3).

**Figure 1:**
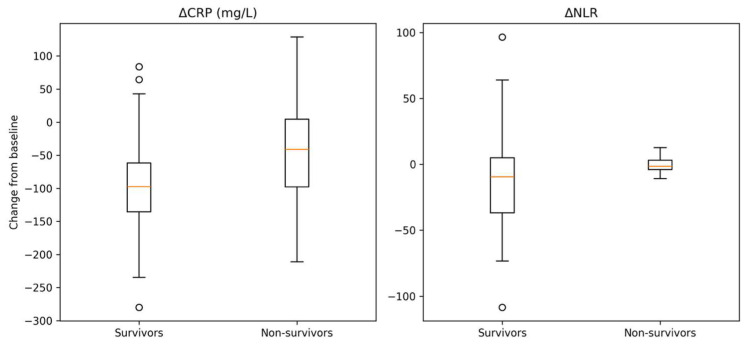
Early changes in inflammatory biomarkers according to in-hospital mortality. Survivors showed a more pronounced decrease in both CRP and NLR compared to non-survivors. Differences were statistically significant (Mann–Whitney U test).

Early changes in inflammatory biomarkers during the first 48–72 h differed between outcome groups. A more pronounced decline in both CRP and NLR was observed in survivors, whereas non-survivors showed a blunted reduction, suggesting persistent inflammatory activity (Table 4). As shown in Figure 1, early changes in inflammatory biomarkers differed according to in-hospital mortality status. Survivors exhibited a marked reduction in both CRP and NLR within the first 48–72 h, whereas non-survivors showed a significantly attenuated decline, particularly for ΔNLR, indicating persistent inflammatory activity and impaired immune resolution.

In regression analysis, several variables were associated with in-hospital mortality in univariate models; however, after adjustment, SOFA score, ΔNLR, and the presence of septic shock remained independent predictors (Table 5).

The predictive performance analysis showed that dynamic markers provided additional prognostic value. ΔNLR demonstrated better discrimination than static inflammatory markers, and the combined model including SOFA score and ΔNLR achieved the highest predictive accuracy (Table 6).

**Figure 2:**
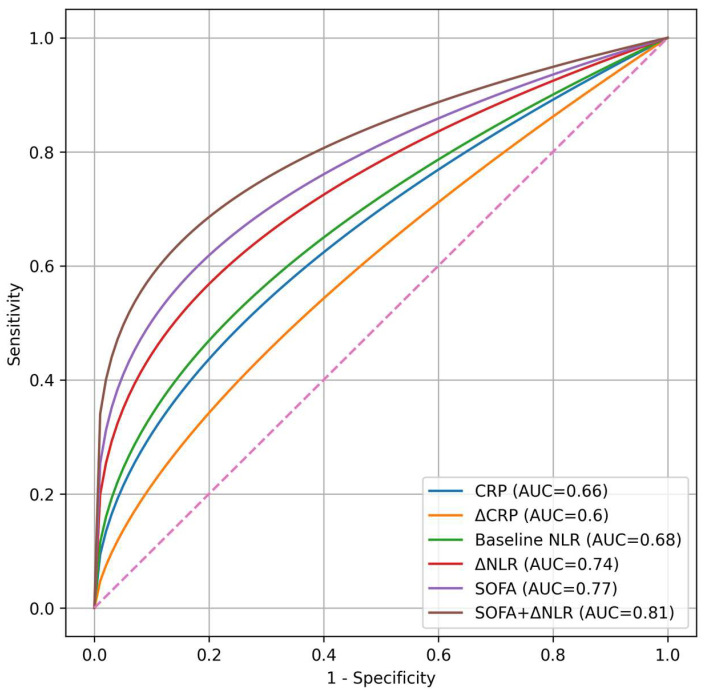
ROC curve analysis of inflammatory biomarkers and clinical scores for predicting in-hospital mortality. Receiver operating characteristic (ROC) curves illustrating the predictive performance of baseline inflammatory markers, dynamic changes, and SOFA score. ΔNLR demonstrated improved discriminative ability compared to static biomarkers, while the combined model (SOFA + ΔNLR) achieved the highest predictive performance. The pink dashed diagonal line indicates the reference line for random prediction (AUC = 0.50), representing the performance expected in the absence of discriminative ability.

Figure 2 illustrates the ROC curve analysis, showing that ΔNLR outperformed static inflammatory biomarkers, while the combined model (SOFA + ΔNLR) achieved the highest predictive accuracy for in-hospital mortality.

Stratification by baseline NLR quartiles showed a progressive increase in mortality, ICU admission, and septic shock rates with higher NLR values, indicating a dose–response relationship between inflammatory burden and adverse outcomes (Table 7).

Correlation analysis confirmed significant associations between inflammatory markers and organ dysfunction severity, with NLR showing the strongest relationship with SOFA score. Early changes in biomarkers were inversely correlated with severity, particularly ΔNLR (Table 8).

**Figure 3:**
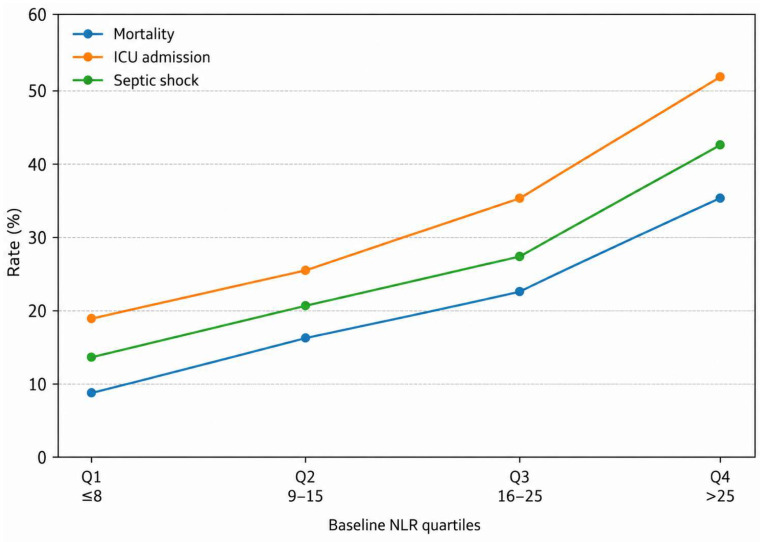
In-hospital outcomes according to quartiles of baseline NLR.

Finally, patients presenting with septic shock at admission had a higher inflammatory profile and more severe disease, along with less pronounced early reductions in inflammatory markers compared to those without shock (Table 9). Moreover, a progressive increase in the rates of mortality, ICU admission, and septic shock was observed across ascending baseline NLR quartiles, with the highest burden of adverse outcomes recorded in patients from the Q4 category (>25), further supporting the association between elevated baseline inflammatory status and unfavorable clinical evolution (Figure 3).

Rates of in-hospital mortality, ICU admission, and septic shock increased progressively across higher quartiles of baseline neutrophil-to-lymphocyte ratio (NLR), suggesting a dose–response relationship between inflammatory burden and adverse clinical outcomes.

## 4. Discussion

The present study demonstrates that early changes in inflammatory biomarkers, particularly the neutrophil-to-lymphocyte ratio (NLR), provide clinically meaningful prognostic information in patients with sepsis, extending beyond what can be captured by baseline values alone. More importantly, our findings show that early ΔNLR represents an independent predictor of in-hospital mortality in a real-world cohort, even after adjustment for disease severity and the presence of septic shock, highlighting its potential clinical utility in early risk stratification. Importantly, rather than representing a limitation, the focus on early biomarker dynamics reflects a clinically critical window in sepsis management, where timely risk stratification may directly influence therapeutic decisions and outcomes.

While previous studies have consistently reported associations between elevated inflammatory markers and adverse outcomes in sepsis, most have focused on baseline measurements [22,23,24]. In our cohort, although CRP, procalcitonin, and NLR were higher in patients with unfavorable outcomes, their predictive value was attenuated after adjustment. This reinforces the limitation of relying on static measurements in a condition characterized by rapid and dynamic pathophysiological changes [25,26].

A key finding of this study is that the trajectory of the inflammatory response within the first 48–72 h provides more robust prognostic information than initial values. Patients who survived showed a marked decline in both CRP and NLR, whereas non-survivors exhibited persistently elevated or only minimally decreasing values. This pattern suggests that the inability to downregulate the inflammatory response early in the disease course is closely linked to poor outcomes.

In this context, ΔNLR emerged as the most informative biomarker. Unlike CRP, which lost significance in the multivariate model, ΔNLR remained independently associated with mortality, supporting the concept that leukocyte-derived indices may better reflect ongoing immune dysregulation. From a pathophysiological perspective, NLR captures both neutrophil-driven inflammation and lymphocyte suppression, two processes that are central to sepsis progression, which may explain its superior performance as a dynamic marker [27,28,29]. This dual representation provides a more nuanced picture of the immune response, reflecting the simultaneous activation of innate immunity and suppression of adaptive immune mechanisms, which are hallmarks of sepsis pathophysiology. As such, leukocyte-derived indices (LDIs) such as NLR offer an integrated view of immune dysregulation that cannot be captured by single inflammatory markers alone [7]. This highlights the novelty of our approach, which lies not in the use of individual biomarkers, but in capturing their early dynamic behavior in a real-world sepsis population.

The predictive performance analysis further strengthens this observation. ΔNLR showed better discrimination compared to baseline inflammatory markers, and when combined with the SOFA score, achieved the highest predictive accuracy, consistent with previous studies showing that the combination of inflammatory biomarkers and clinical severity scores improves prognostic performance in sepsis [1,2]. This finding suggests that ΔNLR adds incremental value to established clinical severity scores, capturing aspects of disease evolution that are not reflected by organ dysfunction alone. Rather than replacing existing tools, ΔNLR appears to complement them, providing a more comprehensive assessment of patient trajectory [30,31].

The progressive increase in mortality and adverse outcomes across NLR quartiles supports the presence of a dose–response relationship between inflammatory burden and clinical severity. However, our data suggest that the persistence of elevated NLR over time may be even more relevant than its baseline value, emphasizing the importance of dynamic assessment. This observation is consistent with previous studies highlighting the prognostic value of dynamic biomarker assessment in sepsis, where early trajectories of inflammatory markers have been shown to better reflect disease evolution and treatment response compared to baseline values alone [13,15]. This distinction is clinically important, as it shifts the focus from initial stratification to continuous monitoring of treatment response.

The observed correlations between inflammatory biomarkers and SOFA score further confirm the well-established link between systemic inflammation and organ dysfunction, as previously described in sepsis pathophysiology studies [32,33]. Among the analyzed markers, NLR showed the strongest association with disease severity, while early reductions in NLR were inversely correlated with SOFA score, suggesting that a rapid normalization of immune parameters may reflect a more favorable clinical course.

Patients presenting with septic shock had both a higher inflammatory burden at admission and a reduced capacity for early biomarker normalization. This finding is consistent with the concept that septic shock is characterized by a more severe and sustained inflammatory response, often accompanied by impaired resolution mechanisms, as described in previous studies on sepsis pathophysiology [34,35,36]. The limited decline in CRP and NLR in these patients further supports the relevance of dynamic biomarkers in identifying those at highest risk.

From a clinical perspective, these findings suggest that monitoring early changes in NLR may represent a simple and accessible tool for improving risk stratification in sepsis. Given its availability and low cost, ΔNLR could be readily integrated into routine practice, particularly in settings where more complex biomarkers are not feasible. Importantly, its added value when combined with the SOFA score supports the development of integrated clinical–biological models that better reflect the dynamic nature of sepsis.

These findings support a shift from static to dynamic biomarker-based risk stratification in sepsis.

### Strengths, Limitations and Future Perspectives

This study has several strengths that support the robustness and clinical relevance of the findings. First, it reflects real-world clinical practice, including a real-world population of patients with sepsis, predominantly elderly, reflecting the typical demographic profile of patients admitted to tertiary care centers. However, the lack of younger age groups should be considered when interpreting the generalizability of the findings. Second, the study design allowed the evaluation of early inflammatory dynamics using routinely available biomarkers, making the findings directly translatable into daily clinical settings. Importantly, the analysis integrated both baseline values and early changes in biomarkers, alongside clinical severity scores, enabling a comprehensive assessment of their relative and combined prognostic value. The consistent performance of ΔNLR across multiple analyses, including regression models and ROC curves, further supports its potential role as a reliable and accessible prognostic marker.

An important aspect of our findings is the differential behavior of inflammatory markers in multivariate analysis. While CRP at admission showed a significant association with mortality in univariate models, this effect was no longer maintained after adjustment for clinical severity and other biomarkers. This likely reflects the fact that CRP is a nonspecific acute-phase reactant that reflects systemic inflammatory activation but does not fully capture the overall inflammatory burden or its complexity, as suggested by its relatively modest correlation with disease severity. In contrast, ΔNLR remained independently associated with in-hospital mortality, suggesting that early changes in leukocyte dynamics provide additional and non-redundant prognostic information. This finding supports the concept that CRP reflects general inflammatory burden, whereas ΔNLR captures the dynamic immune trajectory, integrating both persistent inflammation and lymphocyte suppression.

This distinction highlights a key conceptual difference between static and dynamic biomarkers in sepsis. Baseline CRP reflects the presence and intensity of inflammation at a single time point, whereas ΔNLR captures the early evolution of the host immune response, integrating both the persistence of neutrophil-driven inflammation and the degree of lymphocyte recovery or suppression. As such, ΔNLR may serve as a surrogate marker of immune trajectory rather than inflammatory burden alone.

From a statistical perspective, the loss of significance of CRP in the multivariate model suggests collinearity with clinical severity and other inflammatory parameters, limiting its independent contribution. In contrast, ΔNLR appears to retain unique prognostic information that is not fully explained by baseline severity scores or static biomarkers. This may explain why ΔNLR demonstrated consistent performance across regression and ROC analyses and remained a significant predictor even after adjustment.

However, several limitations should be acknowledged. The retrospective design inherently limits the ability to establish causal relationships and may introduce selection bias. Although inclusion criteria required the availability of serial biomarker measurements, this may have excluded patients with incomplete data, potentially influencing the representativeness of the cohort. Additionally, the timing of follow-up measurements, while restricted to the first 48–72 h, was not fully standardized, which may have introduced variability in the assessment of biomarker dynamics. The incomplete availability of procalcitonin data precluded its inclusion in ROC curve analysis, as missing values could have introduced bias and reduced the reliability of predictive performance estimates. Furthermore, the study was conducted in a single center, which may limit the generalizability of the findings to other healthcare settings with different patient populations or management protocols. Finally, as with any study based on routinely collected data, the potential for unmeasured confounding factors cannot be excluded. The relatively small sample size and single-center design require external validation in larger multicenter cohorts. The robustness of the findings is further supported by consistent results across sensitivity analyses.

Future research should focus on prospective validation of these findings in larger, multicenter cohorts, with standardized timing of biomarker measurements to better define optimal assessment windows. In addition, exploring the integration of ΔNLR into clinical decision-making algorithms or prognostic scores may help determine its practical impact on patient management. Further studies could also evaluate whether biomarker-guided strategies based on early inflammatory dynamics can improve outcomes through earlier therapeutic adjustments. Finally, combining inflammatory biomarkers with emerging molecular or metabolic markers may provide a more refined understanding of sepsis heterogeneity and support the development of personalized approaches to patient care.

## 5. Conclusions

In this cohort of septic patients, baseline inflammatory burden and disease severity were higher in those with unfavorable outcomes; however, their independent predictive value was limited after adjustment. In contrast, early changes in inflammatory biomarkers provided stronger prognostic information. A more pronounced reduction in CRP and especially in NLR within the first 48–72 h was associated with survival, while persistently elevated or minimally decreasing values characterized non-survivors. ΔNLR remained independently associated with in-hospital mortality after adjustment for the SOFA score and septic shock, identifying it as a robust dynamic predictor. Among the evaluated parameters, ΔNLR showed superior predictive performance compared to baseline inflammatory markers, and its combination with the SOFA score achieved the highest accuracy for mortality prediction.

Higher baseline NLR values were associated with a stepwise increase in mortality, ICU admission, and septic shock, suggesting a dose–response relationship between inflammatory burden and adverse outcomes. Patients with septic shock exhibited both higher initial inflammatory levels and a reduced capacity for early biomarker normalization, further supporting the association between persistent inflammation and poor prognosis.

These findings indicate that early inflammatory dynamics, particularly ΔNLR, provide incremental prognostic value over baseline measurements and clinical severity scores in predicting short-term outcomes in sepsis.

## Figures and Tables

**Table 1:** Baseline characteristics of the study cohort.

**Variable**	**Total Cohort (*n* = 168)**
Age, years *	70.47 ± 8.62
Male sex ***	92 (54.8%)
SOFA score at admission *	4.45 ± 2.90
Septic shock at admission ***	38 (22.6%)
Source of infection ***	
• Pulmonary	62 (36.9%)
• Urinary	44 (26.2%)
• Abdominal	24 (14.3%)
• Bloodstream	20 (11.9%)
• Other (skin/soft tissue, CNS, unknown, etc.)	18 (10.7%)
**Comorbidities** ***	
• Diabetes mellitus	58 (34.5%)
• Chronic kidney disease (CKD)	32 (19.0%)
• Chronic heart failure	49 (29.2%)
• COPD	28 (16.7%)
• Active cancer	21 (12.5%)
**Inflammatory biomarkers at admission**	
CRP, mg/L *	174.48 ± 108.52
Procalcitonin (PCT), ng/mL **	2.6 [0.8–8.9] *
WBC, ×10^9^/L *	13.8 ± 6.2
Absolute neutrophils, ×10^9^/L *	11.6 ± 5.8
Absolute lymphocytes, ×10^9^/L *	0.85 ± 0.52
Baseline NLR **	14.8 [8.2–27.6]

Data are presented as * mean ± SD or ** median [IQR] for continuous variables and as *** number (percentage) for categorical variables.

**Table 2:** Baseline characteristics according to in-hospital mortality.

**Variable**	**Survivors (*n* = 132)**	**Non-Survivors (*n* = 36)**
Age, years *	69.8 ± 8.4	73.2 ± 8.7
SOFA score at admission *	3.9 ± 2.3	6.4 ± 3.0
CRP at admission, mg/L *	160.5 ± 98.7	228.4 ± 115.2
Baseline NLR **	13.2 [7.6–24.4]	24.9 [14.1–39.8]

Continuous variables are presented as mean ± SD * or median [IQR] **, as appropriate. Comparisons between groups were performed using the independent samples *t*-test or Mann–Whitney U test, as appropriate.

**Table 3:** Baseline biomarkers and severity scores according to composite adverse outcome.

**Variable**	**No Adverse Outcome (*n* = 112)**	**≥1 Adverse Outcome (*n* = 56)**	***p*** **-Value**
Age, years *	69.8 ± 8.5	71.7 ± 8.7	0.18
SOFA score at admission *	3.6 ± 2.4	6.1 ± 3.1	<0.001
CRP at admission, mg/L *	158.9 ± 101.6	205.3 ± 118.9	0.010
Baseline NLR **	11.9 [6.8–21.7]	23.7 [13.5–36.4]	<0.001
Procalcitonin (PCT), ng/mL **	1.7 [0.6–5.3]	5.1 [1.4–16.2]	<0.001

Continuous variables are presented as * mean ± SD or ** median [IQR], as appropriate. Comparisons between groups were performed using the independent samples *t*-test or Mann–Whitney U test, as appropriate. Categorical variables were compared using the chi-square test or Fisher’s exact test. A two-sided *p*-value < 0.05 was considered statistically significant. Non-normally distributed variable.

**Table 4:** Early inflammatory biomarker dynamics (48–72 h) according to in-hospital mortality.

**Variable**	**Survivors (*n* = 132)**	**Non-Survivors (*n* = 36)**	***p*** **-Value**
**ΔCRP, mg/L (mean ± SD)**	−92.4 ± 71.6	−48.7 ± 83.2	0.021
**ΔNLR (mean ± SD)**	−14.8 ± 28.9	−2.3 ± 7.1	0.004

Continuous variables are presented as mean ± SD. Δ values represent the absolute change between baseline (at admission) and follow-up measurements obtained within 48–72 h; negative values indicate a decrease over time. Comparisons between groups were performed using the independent samples *t*-test or Mann–Whitney U test, as appropriate. A two-sided *p*-value < 0.05 was considered statistically significant.

**Table 5:** Univariate and multivariate logistic regression analysis for in-hospital mortality.

**Variable**	**Univariate OR (95% CI)**	***p*** **-Value**	**Multivariate OR (95% CI)**	***p*** **-Value**
**Age (per 1-year increase)**	0.98 (0.94–1.03)	0.44	0.97 (0.92–1.03)	0.31
**SOFA score (per 1-point increase)**	1.29 (1.12–1.48)	<0.001	1.24 (1.07–1.45)	0.004
**CRP at admission (per 10 mg/L increase)**	1.04 (1.01–1.07)	0.009	1.02 (0.99–1.06)	0.11
**ΔCRP (per 10 mg/L change)**	0.97 (0.94–1.00)	0.048	0.99 (0.95–1.03)	0.62
**Baseline NLR (per 1-unit increase)**	1.03 (1.00–1.05)	0.032	1.02 (0.99–1.05)	0.14
**ΔNLR (per 1-unit change)**	0.89 (0.83–0.95)	0.001	0.91 (0.84–0.98)	0.015
**Septic shock at admission**	3.21 (1.48–6.95)	0.003	2.74 (1.18–6.36)	0.019

Results are presented as odds ratios (ORs) with 95% confidence intervals (CIs). Univariate and multivariate logistic regression analyses were performed to identify predictors of in-hospital mortality. Δ values represent the absolute change between baseline (at admission) and follow-up measurements obtained within 48–72 h.

**Table 6:** ROC curve analysis for predicting in-hospital mortality.

**Parameter**	**AUC (95% CI)**	**Optimal Cut-Off**	**Sensitivity (%)**	**Specificity (%)**
**CRP at admission (mg/L)**	0.66 (0.56–0.76)	>190	61	64
**ΔCRP (mg/L)**	0.60 (0.50–0.70)	>−70	58	57
**Baseline NLR**	0.68 (0.58–0.78)	>18	64	66
**ΔNLR**	0.74 (0.65–0.83)	>−5	72	69
**SOFA score**	0.77 (0.68–0.86)	≥5	75	71
**Model: SOFA + ΔNLR**	**0.81 (0.73–0.89)**	—	78	76

AUC values are presented with 95% confidence intervals (CIs). Optimal cut-off values were determined using the Youden index. Sensitivity and specificity are reported for each parameter. Δ values represent the absolute change between baseline (at admission) and follow-up measurements obtained within 48–72 h.

**Table 7:** Association between NLR quartiles and adverse clinical outcomes in patients with sepsis.

**NLR Quartile**	**NLR Range**	**Mortality *n* (%)**	**ICU Admission *n* (%)**	**Septic Shock *n* (%)**
Q1 (lowest)	≤8	4 (9.5%)	8 (19.0%)	6 (14.3%)
Q2	9–15	7 (16.7%)	11 (26.2%)	9 (21.4%)
Q3	16–25	10 (23.8%)	15 (35.7%)	12 (28.6%)
Q4 (highest)	>25	15 (35.7%)	22 (52.4%)	18 (42.9%)

Data are presented as number (percentage). Patients were stratified according to quartiles of baseline neutrophil-to-lymphocyte ratio (NLR), defined based on its distribution in the study cohort.

**Table 8:** Correlation between inflammatory biomarkers and organ dysfunction severity (SOFA score at admission).

**Biomarker**	**Correlation with SOFA (r)**	***p*** **-Value**
CRP at admission	0.28	<0.001
Procalcitonin (PCT)	0.34	<0.001
Baseline NLR	0.37	<0.001
ΔCRP (48–72 h)	−0.19	0.015
ΔNLR (48–72 h)	−0.31	<0.001

Correlation coefficients (r) were calculated using Spearman’s rank correlation. Δ values represent the absolute change between baseline (at admission) and follow-up measurements obtained within 48–72 h.

**Table 9:** Baseline inflammatory biomarkers and early dynamics according to presence of septic shock at admission.

**Variable**	**No Septic Shock (*n* = 130)**	**Septic Shock (*n* = 38)**	***p*** **-Value**
**SOFA score (mean ± SD)**	3.7 ± 2.2	7.2 ± 2.9	<0.001
**CRP at admission (mg/L) (mean ± SD)**	166.5 ± 101.3	208.9 ± 121.7	0.018
**Procalcitonin (ng/mL) (median [IQR])**	1.9 [0.7–6.2]	8.6 [2.5–22.4]	<0.001
**Baseline NLR (mean ± SD)**	16.9 ± 21.8	28.7 ± 43.6	0.041
**ΔCRP (mg/L) (mean ± SD)**	−86.4 ± 66.1	−58.3 ± 72.8	0.029
**ΔNLR (mean ± SD)**	−14.8 ± 28.5	−3.1 ± 6.4	0.004

Continuous variables are presented as mean ± SD or median [IQR], as appropriate. Comparisons between groups were performed using the independent samples *t*-test or Mann–Whitney U test, as appropriate. Δ values represent the absolute change between baseline (at admission) and follow-up measurements obtained within 48–72 h; negative values indicate a decrease over time. A two-sided *p*-value < 0.05 was considered statistically significant.

## Data Availability

The original contributions presented in this study are included in the article. Further inquiries can be directed to the corresponding authors.
